# A Simple in-vitro Test for Assessing the Sensitivity of Lymphocytes to Chlorambucil

**DOI:** 10.1038/bjc.1971.62

**Published:** 1971-09

**Authors:** Sylvia D. Lawler, Kusum P. Lele, C. R. Pentycross

## Abstract

The sensitivity of lymphocytes to chlorambucil has been assessed by a simple *in-vitro* test which has been applied to the cells of normal controls and of patients with chronic lymphocytic leukaemia. The degree of sensitivity varied amongst the normal controls and *in-vitro* resistance of the lymphocytes in the patients was sometimes found in the absence of *in-vivo* experience of the drug. Resistance *in-vitro* tended to be associated with very high total peripheral blood lymphocyte counts but not with the age of the patient. Where the information was available the *in-vitro* sensitivity test agreed with the results of biochemical estimations of drug resistance and with the clinical responses to the drug. It is suggested that this test may have applications in patient management.


### A SIMPLE *IN-VITRO* TEST FOR ASSESSING THE SENSITIVITY OF LYMPHOCYTES TO CHLORAMBUCIL

SYLVIA D. LAWLER, KUSUM P. LELE AND C. R. PENTYCROSS

*from the Department of Clinical Research, Royal Marsden Hospital and Institute of Cancer Research, London, S.W.3*

Received for publication June 18, 1971

**SUMMARY.—The sensitivity of lymphocytes to chlorambucil has been assessed by a simple *in-vitro* test which has been applied to the cells of normal controls and of patients with chronic lymphocytic leukaemia. The degree of sensitivity varied amongst the normal controls and *in-vitro* resistance of the lymphocytes in the patients was sometimes found in the absence of *in-vivo* experience of the drug. Resistance *in-vitro* tended to be associated with very high total peripheral blood lymphocyte counts but not with the age of the patient. Where the information was available the *in-vitro* sensitivity test agreed with the results of biochemical estimations of drug resistance and with the clinical responses to the drug. It is suggested that this test may have applications in patient management.**

In a study of the karyotypes of circulating lymphocytes from cases of chronic lymphocytic leukaemia it was found that a minor population of cells in patients treated with chemotherapy had an increased number of chromosomal rearrangements, as compared with normal controls and untreated patients (Lawler *et al.*, 1968). The question arose whether the rearranged cells in the treated patients should be attributed to the action of the drugs or to a fundamental disorder of a minor population of cells in those patients who required treatment. The drugs used were alkylating agents, such as chlorambucil, sometimes combined with prednisolone. The patients studied had not been irradiated.

Alkylating agents are known to react with deoxyribonucleic acid and therefore rearrangements of the chromosomes are a theoretical possibility. Nevertheless it must be appreciated that when these drugs are successful in controlling the total leucocyte count in patients with chronic lymphocytic leukaemia most of the cells that are eliminated are undoubtedly in interphase and they probably die without going into a subsequent division cycle.

*In-vitro* experiments were designed to compare the effect of chlorambucil on lymphocytes stimulated with mitogenic agents from both normal controls and patients with chronic lymphocytic leukaemia. When examining the control experiments which contained no mitogenic agent, it became apparent that the simple procedure of recording cell survival in lymphocyte populations exposed to chlorambucil for several days could be used as a measure of their sensitivity to the drug.

### MATERIALS AND METHODS

The experiments were set up with the peripheral blood lymphocytes obtained from 12 patients (9 males and 3 females) with chronic lymphocytic leukaemia

(CLL) and four normal control subjects (2 males and 2 females). From each individual 20 to 30 ml. of blood were collected into phenol-free heparin (12·5 units/ml.) and sedimented upright at 37° C. for ½–1½ hours. In most of the patients almost all the cells in the supernatant plasma were lymphocytes, but in one patient and in all the controls contamination with other types of white cell necessitated their removal. This was done by adding medium TC 199 to the supernatant plasma in the proportion of 1 : 1 and incubating at 37° C. for 30 minutes in a medicine bottle. Most of the granulocytes and monocytes became attached to the flat surface of the bottle, the lymphocytes remaining in suspension. Before setting up the cultures the lymphocytes were always washed in TC 199 with heparin (10 units/ml.). The culture medium consisted of TC 199 and human AB serum in the proportions of 4 : 1. The final lymphocyte concentration was approximately 1000/mm$^3$.

*Preparation and standardization of the dose of chlorambucil*

Fifty mg. of chlorambucil (Burroughs Wellcome & Co.) were dissolved in 1 ml. of " acid-alcohol " and 9 ml. of propylene glycol. From this stock solution several serial dilutions were made with the culture medium. In the pilot experiments the effect of different doses of the drug were studied on the lymphocytes from the normal individuals and 0·5 μg./ml. was found to be the suitable dose for assessing relative sensitivities of lymphocytes to the drug.

*Unstimulated cultures*

Lymphocytes from some patients were exposed to 0·7 μg./ml. and 1·0 μg./ml. of drug in addition to the standard dose of 0·5 μg./ml. Drug diluent, without drug, was added to some cultures to ensure that the diluent was innocuous. The cells were incubated at 37° C. for 120 hours. Every 24 hours 0·5 ml. of cell suspension from each culture was centrifuged and smears were made from the cell pellet, air-dried, fixed in methyl alcohol for 10 minutes, and stained with May-Grünwald and Giemsa.

*Stimulated cultures*

Mitogens were added to cell cultures from some patients. In the case of both phytohaemagglutinin (PHA) and pokeweed (PWM) the amount of mitogen added was 0·1 ml. reconstituted material per 10 ml. culture. For each experiment with mitogens, six cultures were set up thus:

1. Cells with PHA (Burroughs Wellcome & Co.).
2. Cells with PWM (Grand Island Biological Co.).
3. Cells with PHA added 10 minutes after chlorambucil.
4. Cells with PWM added 10 minutes after chlorambucil.
5. Cells with PHA, ab initio, drug added at 72 hours.
6. Cells with PWM, ab initio, drug added at 72 hours.

All six cultures were incubated at 37° C. for a total of 118 hours and sampled at 24, 48, 72, 96 and 118 hours. In cultures numbers 3 and 4 chlorambucil was added at 37° C.

FIG. 1.—Lymphocytes from a patient (Case 5, 2nd test) with chronic lymphocytic leukaemia, showing marked sensitivity to three doses of chlorambucil *in vitro* in a 120 hour culture.

FIG. 2.—Lymphocytes from a patient (Case 10) with chronic lymphocytic leukaemia, showing marked resistance to three doses of chlorambucil *in vitro* in a 120 hour culture.

*Sensitivity scores*

Cells were scored as " dead " if they were pyknotic. Smudge cells were likewise scored as " dead ". For the purposes of these experiments cells not falling into either of the above categories were regarded as " surviving ".

A score of sensitivity was obtained by the difference between the percentage of cells surviving in control cultures and in those containing chlorambucil.

| | | |
|---|---|---|
| Sensitive | (S) | = 30+ |
| Moderately sensitive | (MS) | = 15–29 |
| Resistant | (R) | = 0–14 |

### RESULTS

Fig. 1 and 2 show graphs of the cell survival patterns of lymphocytes from two patients measured by daily sampling over a period of 120 hours at three different concentrations of chlorambucil.

In Fig. 1 a marked degree of sensitivity is evident in the culture treated with a concentration of 0·5 μg./ml. of chlorambucil. The higher concentrations are associated with even higher scores of sensitivity (Case 5, 2nd test, Table II). The drug diluent is seen to be innocuous.

Fig. 2 shows a typical graph of survival of lymphocytes that are resistant to chlorambucil (Case 10, Table II).

Table I shows the results of the sensitivity tests on four normal subjects. The subjects are arranged according to the sensitivity of their lymphocytes to 0·5 μg./ml. of drug at 120 hours. It will be observed that the lymphocytes from two females and one male were classified as sensitive whereas those from the remaining male were only moderately sensitive.

TABLE I.—*Sensitivity Tests on the Lymphocytes of Normal Controls*

| | | | 120 hours | |
|---|---|---|---|---|
| No. | Age | Sex | Concentration of the drug | Sensitivity* score |
| 1 | 26 | F | 0·5 μg./ml. | S (54) |
| 2 | 24 | F | 0·5 μg./ml. | S (47) |
| 3 | 28 | M | 0·5 μg./ml. | S (30) |
| 4 | 26 | M | 0·5 μg./ml. | M.S (18) |

* Sensitivity score given by % lymphocytes surviving in control cultures *minus* % lymphocytes surviving in cultures treated with chlorambucil.

S = Sensitive 30+. MS = Moderately Sensitive 15–29. R = Resistant 0–14.

Table II shows the complete results on the 12 patients also arranged according to their lymphocyte sensitivity to 0·5 μg./ml. of drug at 120 hours. The lymphocytes from Case No. 1, 2 and 3 were classified as sensitive. Case No. 4 had only moderately sensitive lymphocytes but she had never been treated with chlorambucil. Case No. 5 showed variation in sensitivity at different times. In the first test the lymphocytes were resistant to a concentration of 0·5 μg./ml. but moderately sensitive to the higher concentrations. In the second test the lymphocytes were sensitive to 0·5 μg./ml: the patient has now responded clinically to the drug.

All the other cases were classified as resistant: of these only Case 6 had not been treated with chlorambucil at any time. When the concentration of chlorambucil in the cultures was increased to 0·7 μg./ml. or 1·0 μg./ml., the sensitivity scores were still in the resistant range.

TABLE II.—*Sensitivity Tests on Lymphocytes from Patients with Chronic Lymphocytic Leukaemia*

| Case No. | Age | Sex | Lymphocytes per cu.mm. in peripheral blood | Conc. of the drug (μg./ml.) | Sensitivity* score at 120 hours | Date of test | Clinical details |
|---|---|---|---|---|---|---|---|
| (1) AJW | 69 | M | 9800 | 0·5 | S (82) | 15.6.70 | Diagnosed 2½ years (plus ca. left ear). No anti-leukaemic drugs given. |
| (2) KE | 56 | F | 870 | 0·5<br>0·7 | S (48)<br>S (53) | 2.11.70 | Diagnosed 22 months. Has had prednisolone and chlorambucil. On therapy on the day of the experiment. |
| (3) JBH | 69 | M | 34000 | 0·5<br>0·7<br>1·0 | S (31)<br>S (40)<br>S (46) | 9.11.70 | Diagnosed 16 months. Treated with chlorambucil for 5 months —no treatment for 2 months |
| (4) DG | 82 | F | 64600 | 0·5<br>1·0 | M.S (19)<br>M.S (18) | 6.4.70 | Diagnosed 2½ years. Vulvectomy for Ca. vulva in 1962. No anti-leukaemic drugs given. |
| (5) GS | 66 | M | 26878 | 0·5<br>0·7<br>1·0 | R (2)<br>M.S (23)<br>M.S (24) | 9.11.70 | Diagnosed 2 years and 8 months. Has been treated with prednisolone and chlorambucil. No therapy for 6 months. |
| | | | 75716 | 0·5<br>0·7<br>1·0 | S (32)<br>S (49)<br>S (64) | 1.2.71 | |
| (6) KR | 69 | F | 305550 | 0·5 | R (10) | 24.8.70 | Diagnosed 2 months. No treatment given. |
| (7) IF | 70 | M | 7524 | 0·5 | R (8) | 28.9.70 | Diagnosed 23 years. Has been treated with chlorambucil. No treatment for last 11 months. |
| (8) RER | 72 | M | 9350 | 0·5<br>0·7<br>1·0 | R (7)<br>R (1)<br>R (14) | 16.11.70 | Diagnosed 9 months. Has had chlorambucil, prednisolone and blood transfusions—on no therapy for last 6 months. |
| (9) AA | 57 | M | 354050 | 0·5<br>0·7<br>1·0 | R (4)<br>R (6)<br>R (14) | 8.3.71 | Diagnosed 2 months. Has been treated with prednisolone and chlorambucil and had blood transfusions. Was on prednisolone and chlorambucil at the time of the experiment. |
| (10) GN | 48 | M | 116840 | 0·5<br>0·7<br>1·0 | R (2)<br>R (7)<br>R (11) | 24.11.70 | Diagnosed 4½ years. Treated with chlorambucil and steroids for 3½ years. No treatment for the last year. |
| (11) RP | 53 | M | 212000 | 0·5 | R (0) | 3.8.70 | Diagnosed 9 years. Has been treated with chlorambucil, prednisolone and blood transfusions—was on prednisolone at the time of the experiment. |
| (12) WLC | 70 | M | 53000 | 0·5<br>0·7 | R (0)<br>R (0) | 12.10.70 | Diagnosed 7 years. Has had chlorambucil, prednisolone, cyclophosphamide and blood transfusions. No specific treatment for the last 4 months. Splenectomy in 1966. |

* See footnote to Table I.

FIG. 3.—Lymphocytes from a patient (Case 6) with chronic lymphocytic leukaemia, showing marked resistance to 0·5 μg./ml. of chlorambucil in the presence of mitogens.

FIG. 4.—Lymphocytes from a patient (Case 1) with chronic lymphocytic leukaemia, showing a difference in sensitivity according to the time at which drug and mitogens were added.

Fig. 3 and 4 illustrate the effects of the addition of mitogens on the action of the drug on lymphocyte cultures which were sampled daily up to 118 hours.

Fig. 3 demonstrates that the addition of mitogens to resistant lymphocytes does not alter their sensitivity (Case No. 6, Table II).

Fig. 4 shows the effect of the addition of mitogens to lymphocytes that are sensitive to chlorambucil *in-vitro*. If the drug is present *ab initio*, the addition of mitogen 10 minutes afterwards does not alter the sensitivity pattern of the lymphocytes. On the other hand, if the drug is added three days after the mitogen, then at 5 days the lymphocytes are apparently resistant to chlorambucil. However it must be appreciated that the cells have only been exposed to drug for 2 days (Case No. 1, Table II).

### DISCUSSION

Whilst assessing drug-induced chromosomal damage *in vitro*, it became apparent that the sensitivity to chlorambucil of the lymphocytes of normal controls and patients with chronic lymphocytic leukaemia could be estimated by a simple *in-vitro* test. In this test lymphocytes that had not been exposed to mitogens were treated with chlorambucil, the standard dose being 0·5 $\mu$g. per ml. of culture medium. The lymphocytes were incubated at 37° C. and the number of surviving cells was counted in samples taken daily over a period of 5 days.

The most clear-cut differences between sensitive and resistant populations of lymphocytes were obtained after 5 days' incubation. The test cannot be used with lymphocytes that are sensitive to the manipulations preceding incubation because they give a low score of surviving cells in the absence of the drug.

In our experience a more reliable result is given by reading the tests from stained smears than by the use of the trypan blue dye exclusion test of cell viability. Degenerating cells are "lost" from wet preparations but they show up in smears as smudges in which chromatin is identifiable. The stained smears also have the advantage of providing a permanent record of the test.

The sensitivity of lymphocytes to chlorambucil varies in normal controls. Using our criteria for scoring, the lymphocytes of one of the normal controls were only moderately sensitive to the standard dose of the drug: a score in the sensitive range was obtained by increasing the concentration of chlorambucil to 1 $\mu$g. per ml. of culture. Two patients had not been treated with chlorambucil, one (Case 4), had lymphocytes which were only moderately sensitive to the drug, the other (Case 6), had cells that were resistant. Thus resistance of lymphocytes to chlorambucil in the *in-vitro* test amongst patients with chronic lymphocytic leukaemia can be present in the absence of *in vivo* experience of the drug. This is in accord with the observation of " natural " resistance of the disease process to chlorambucil therapy (Larionov, 1962).

The other patients with resistant lymphocytes (Cases 7, 8, 9, 10, 11 and 12) had all been treated with chlorambucil at some time during the course of their disease. The lymphocytes of these patients were still resistant when exposed to concentrations of drug up to 1 $\mu$g. per ml. of culture. Most of the patients whose lymphocytes showed resistance had very high total lymphocyte counts, whilst patients whose lymphocytes were sensitive included those with both high and low total counts. Drug resistance was not necessarily a feature of old age.

The experiments in which the lymphocytes were exposed to both mitogenic agents and chlorambucil showed that the classification of the cells as resistant or sensitive remained the same. Perhaps this is not surprising in view of the fact that only a minority of the cell population responds to mitogenic stimulation in chronic lymphocytic leukaemia. There is, however, a suggestion in the data that mitogens may exert a protective effect. For example, the cells of Case No. 1, (Fig. 4) showed a higher survival rate if they were exposed to mitogens for 3 days before the addition of the drug, as compared with the cultures in which drug was added first followed by mitogen. This result can be questioned because all the experiments were terminated at 5 days. It is a tenable argument that in order to make a valid comparison the experiment in which the drug was added 3 days after the mitogen should not have been terminated until 7 days.

Drs. K. Harrap and Bridget Hill, working at the Chester Beatty Research Institute are investigating the problem of drug resistance to alkylating agents by a biochemical method. They have measured the capacity of the lymphocytes to degrade chlorambucil in four cases that we have also studied (Hill, 1968; Harrap and Hill, personal communication, 1971). Case 4 was found by us to have lymphocytes that were only moderately sensitive to chlorambucil, and in the biochemical test the cells were found to be sensitive but with " some slight degradative ability ". Case 11 was scored as sensitive biochemically in 1968, but had become resistant by 1969, the lymphocytes were resistant in our test in 1970. Case 9 showed resistance in both the biochemical and the *in-vitro* culture tests, both tests being done within 1 month of each other. The behaviour of the lymphocytes of Case 5 is particularly interesting since the cells have been found to be either sensitive or resistant at different times by both methods. Thus the results obtained by the two methods are in agreement.

The assessment of the accord between the *in-vitro* culture test of sensitivity and the response of the patients to chlorambucil is complicated by the fact that some of the patients have not been treated with the drug, whilst others have had other forms of therapy concomitantly. Where it has been possible to make a clinical judgement as to whether the patient was responding to chlorambucil at a particular time, there is agreement with the results of the *in-vitro* test (Galton, personal communication, 1971).

Chlorambucil undoubtedly can kill cells in interphase, and in this respect differs from other cytotoxic drugs. The simple *in-vitro* test that we have happened upon exploits this activity. We hope that the test may have applications in patient management.

We thank Dr. D. A. G. Galton for permission to study his patients and acknowledge the help of the Departments of Clinical Pathology, Medical Art and Photography, of the Royal Marsden Hospital, London. We are grateful to Drs. L. Cobb and B. R. Reeves for constructive criticisms of the manuscript.

One of us (K.P.L.) thanks the Board of Governors of the Royal Marsden Hospital for a Gordon Jacobs Fellowship.

C.R.P. gratefully acknowledges the receipt of a grant from the Joseph Strong Frazer Trust in support of his work.

### REFERENCES

HILL, B. T.—(1968) Ph.D. Thesis (London).

LARIONOV, L. F.—(1962) *Cancer Chemother. Abstr.*, **3**, 55.

LAWLER, S. D., PENTYCROSS, C. R. AND REEVES, B. R.—(1968) *Br. med. J.*, **iv**, 213.


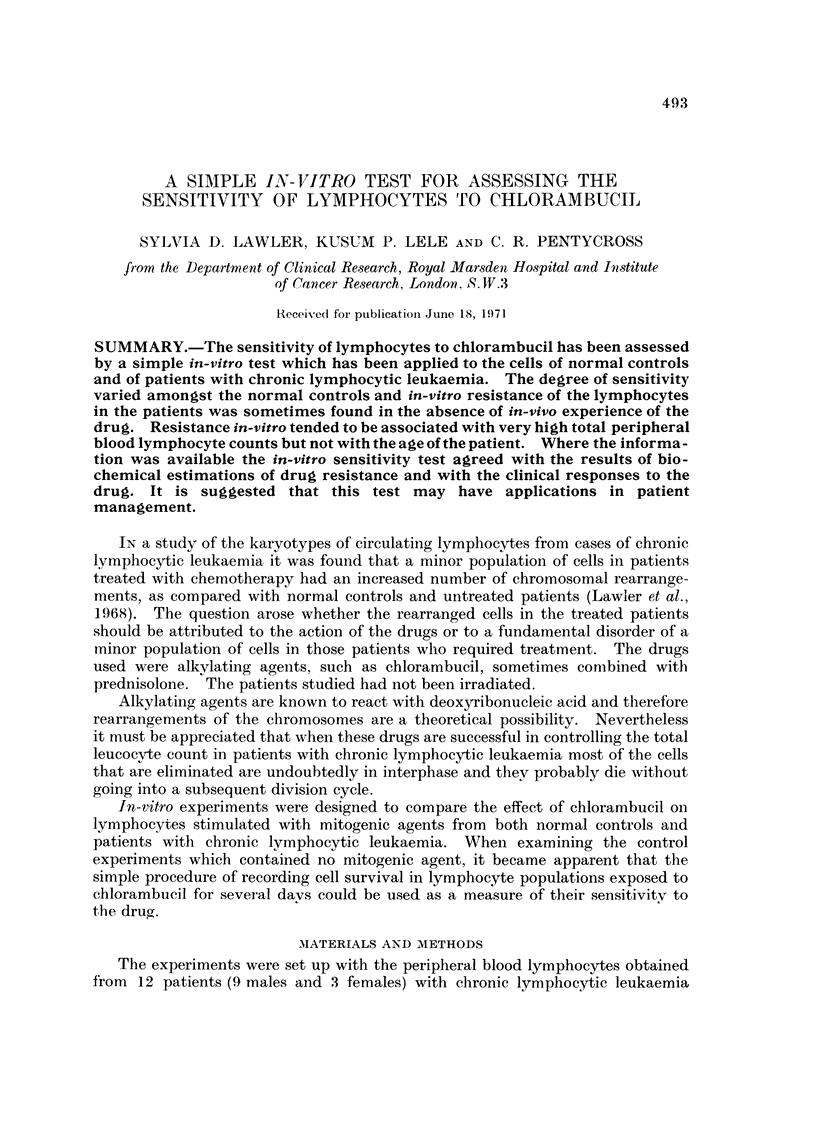

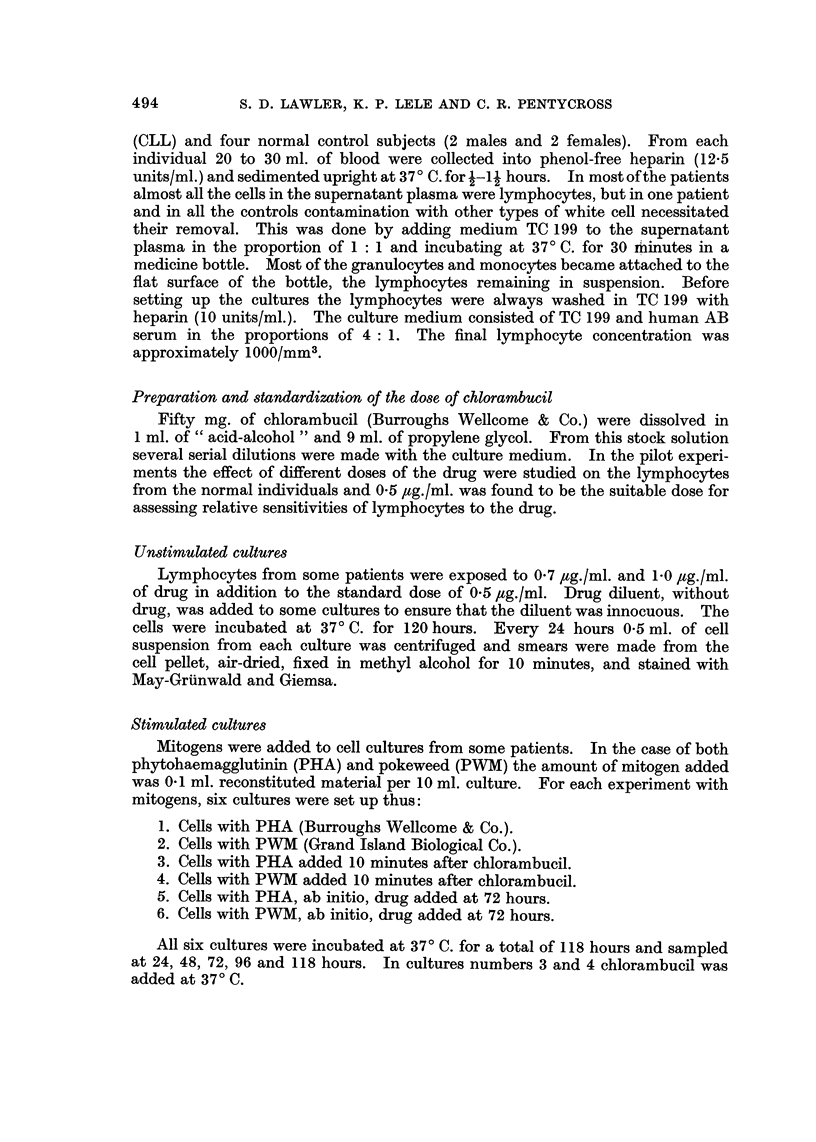

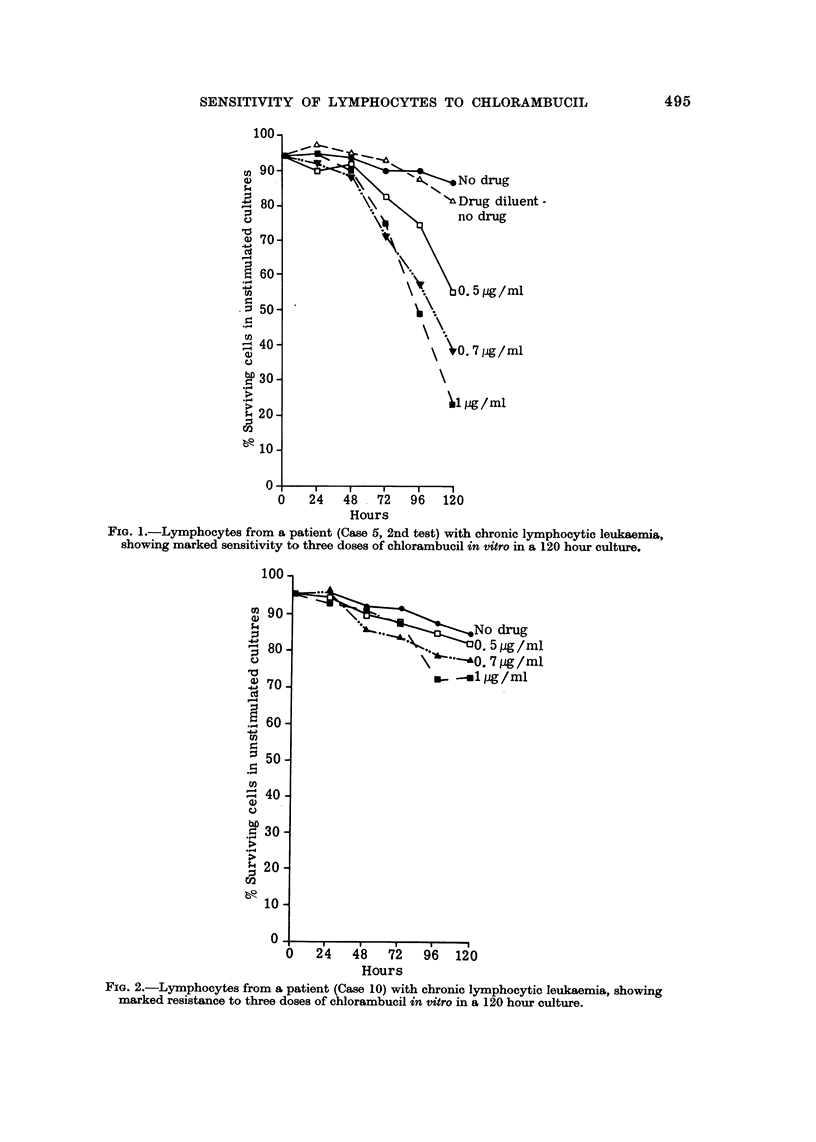

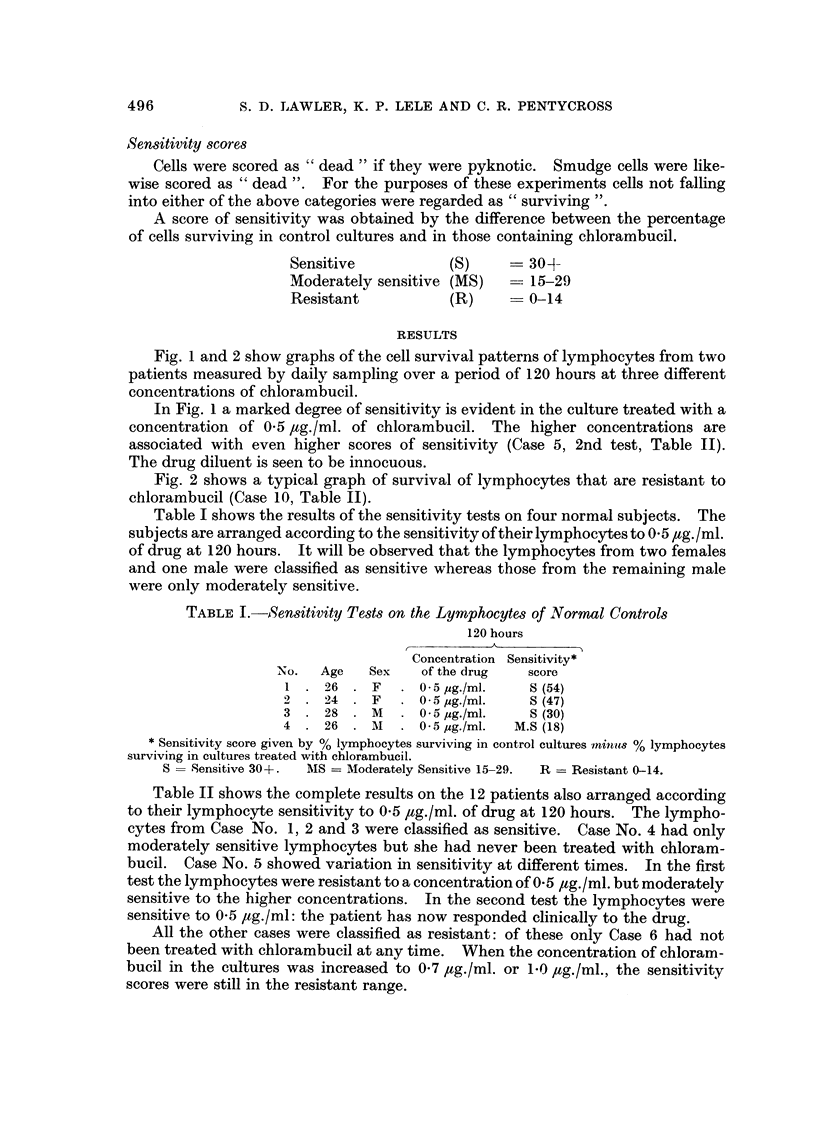

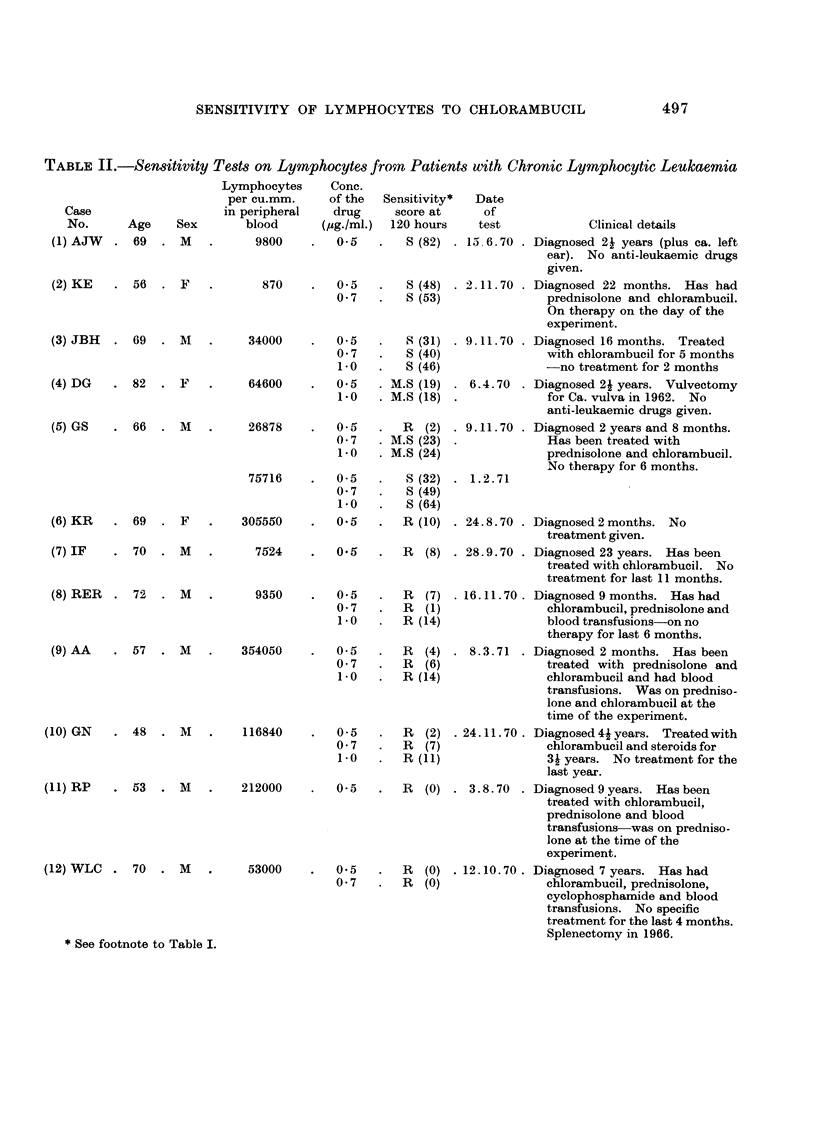

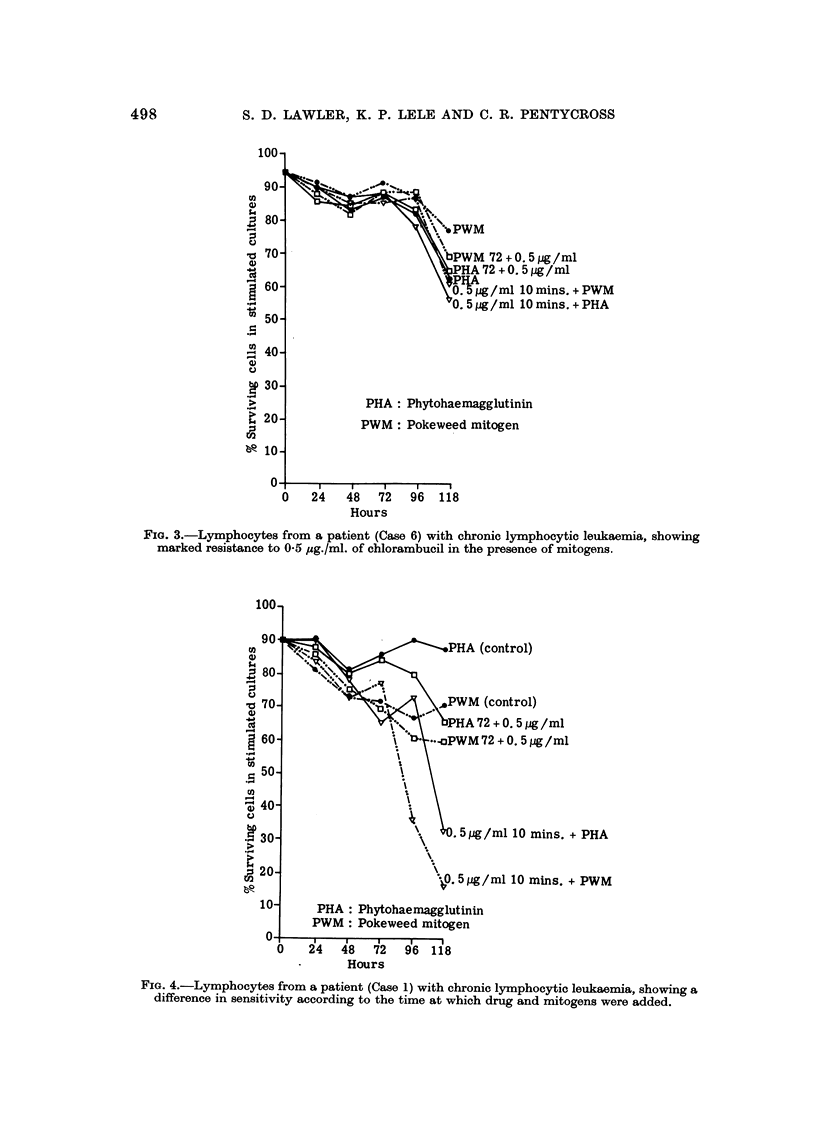

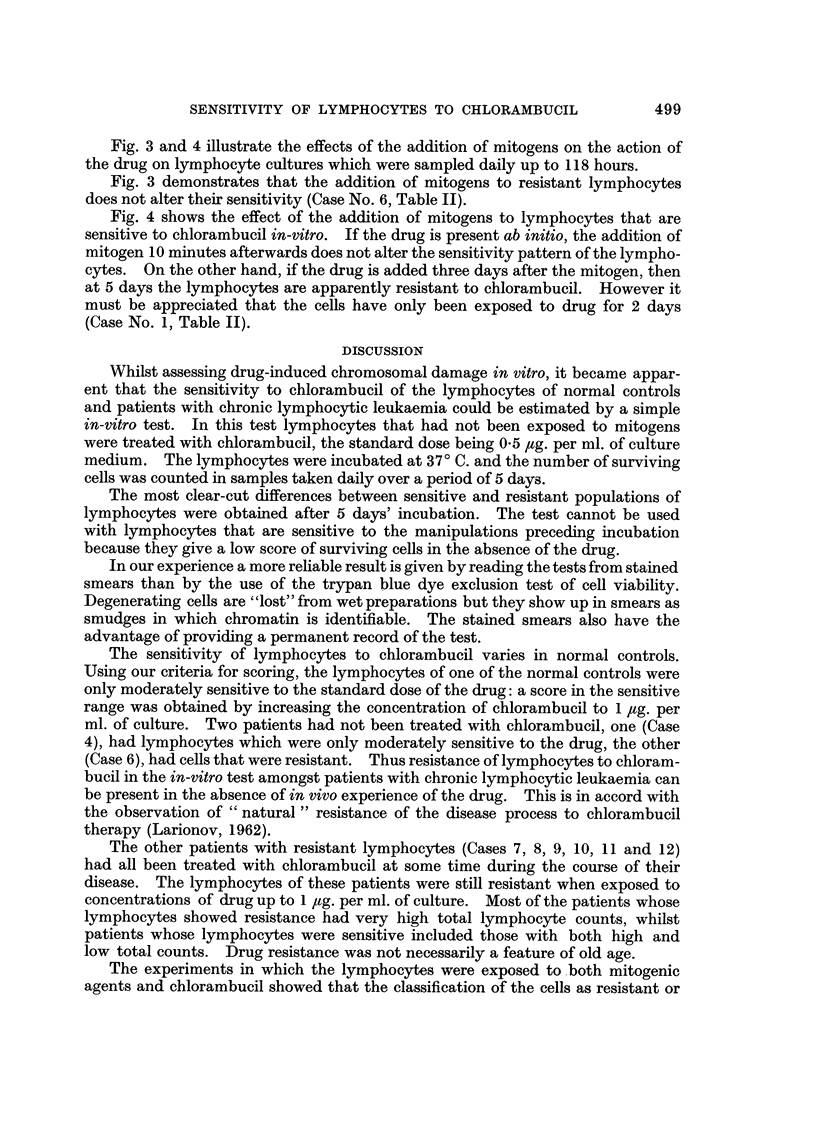

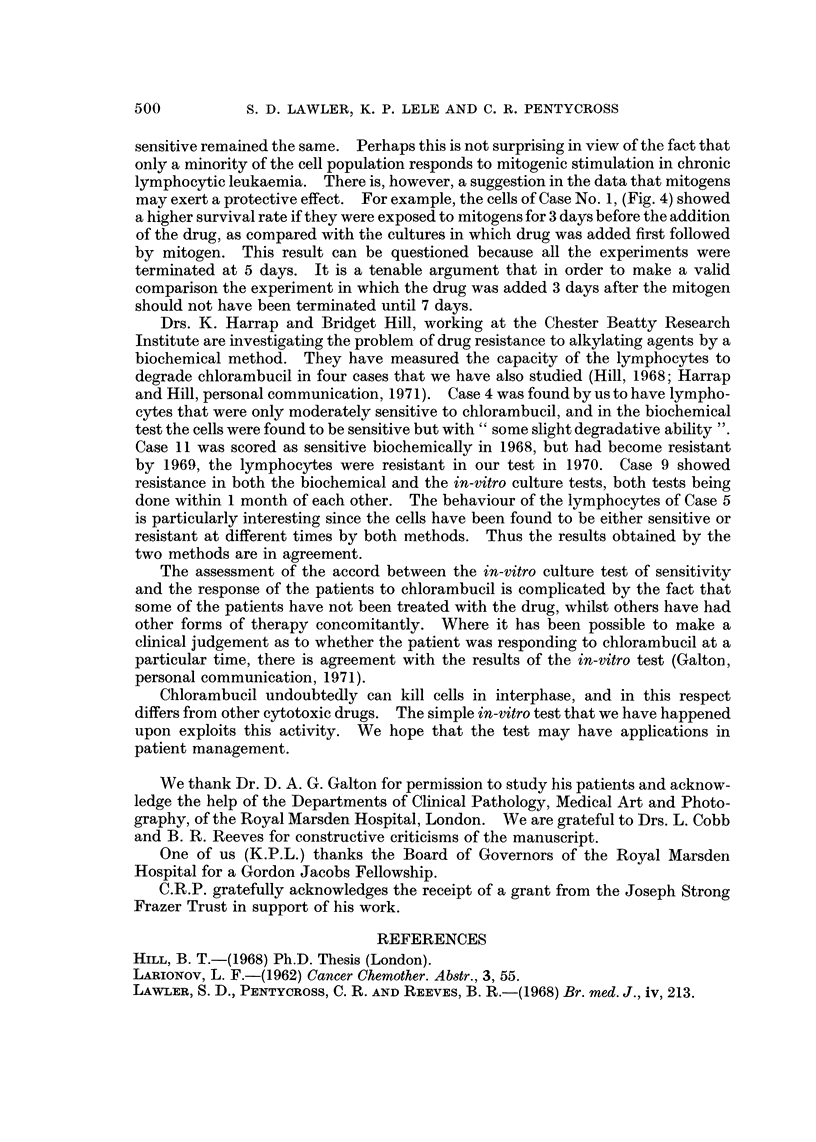


## References

[OCR_00677] Lawler S. D., Pentycross C. R., Reeves B. R. (1968). Chromosomes and transformation of lymphocytes in lymphoproliferative disorders.. Br Med J.

